# The Importance of Body Weight for the Dose Response Relationship of Oral Vitamin D Supplementation and Serum 25-Hydroxyvitamin D in Healthy Volunteers

**DOI:** 10.1371/journal.pone.0111265

**Published:** 2014-11-05

**Authors:** John Paul Ekwaru, Jennifer D. Zwicker, Michael F. Holick, Edward Giovannucci, Paul J. Veugelers

**Affiliations:** 1 School of Public Health, University of Alberta, Edmonton, Alberta, Canada; 2 School of Public Policy, University of Calgary, Calgary, Alberta, Canada; 3 Section of Endocrinology, Nutrition and Diabetes, Department of Medicine, Boston University School of Medicine, Boston, Massachusetts, United States of America; 4 Harvard School of Public Health, Departments of Nutrition and Epidemiology, Boston, Massachusetts, United States of America; University of Southampton, United Kingdom

## Abstract

Unlike vitamin D recommendations by the Institute of Medicine, the Clinical Practice Guidelines by the Endocrine Society acknowledge body weight differentials and recommend obese subjects be given two to three times more vitamin D to satisfy their body's vitamin D requirement. However, the Endocrine Society also acknowledges that there are no good studies that clearly justify this. In this study we examined the combined effect of vitamin D supplementation and body weight on serum 25-hydroxyvitamin (25(OH)D) and serum calcium in healthy volunteers. We analyzed 22,214 recordings of vitamin D supplement use and serum 25(OH)D from 17,614 healthy adult volunteers participating in a preventive health program. This program encourages the use of vitamin D supplementation and monitors its use and serum 25(OH)D and serum calcium levels. Participants reported vitamin D supplementation ranging from 0 to 55,000 IU per day and had serum 25(OH)D levels ranging from 10.1 to 394 nmol/L. The dose response relationship between vitamin D supplementation and serum 25(OH)D followed an exponential curve. On average, serum 25(OH)D increased by 12.0 nmol/L per 1,000 IU in the supplementation interval of 0 to 1,000 IU per day and by 1.1 nmol/L per 1,000 IU in the supplementation interval of 15,000 to 20,000 IU per day. BMI, relative to absolute body weight, was found to be the better determinant of 25(OH)D. Relative to normal weight subjects, obese and overweight participants had serum 25(OH)D that were on average 19.8 nmol/L and 8.0 nmol/L lower, respectively (P<0.001). We did not observe any increase in the risk for hypercalcemia with increasing vitamin D supplementation. We recommend vitamin D supplementation be 2 to 3 times higher for obese subjects and 1.5 times higher for overweight subjects relative to normal weight subjects. This observational study provides body weight specific recommendations to achieve 25(OH)D targets.

## Introduction

Vitamin D has been shown to benefit bone health, to prevent rickets, osteomalacia and symptomatic hypocalcaemia, and to reduce the burden of other specific diseases [1]–[5]. To reduce burden of disease, various institutions recommend defined amounts of vitamin D intake [6]–[8]. The established proxy for vitamin D status, however, is serum 25-hydroxyvitamin D (25(OH)D) [9], [10]. This proxy has been used for definitions of vitamin D deficiency (for example, serum 25(OH)D levels below 50 nmol/L), vitamin D insufficiency (serum 25(OH)D levels between 50 and 75 nmol/L), and vitamin D toxicity (serum 25(OH)D levels exceeding 500 nmol/L) [7], though these definitions are not established. With recommendations based on vitamin D intake and serum 25(OH)D as the best proxy for nutritional status, a good quantification of the dose response relationship between vitamin D intake and serum 25(OH)D is essential. However, this dose response relationship is currently not well documented, particularly not for a wider range and including high levels of vitamin D supplementation.

The Recommended Dietary Allowance (RDA) is the nutrient intake considered to be sufficient to meet the requirements of 97.5% of healthy individuals. The RDA for vitamin D, 600 IU day for individuals 1 to 70 years of age and 800 IU per day for those above the age of 70 years [8]. Although differences in serum 25(OH)D by body mass index (BMI) and by absolute body weight have been reported [11]–[19], the RDA does not consider either. The Clinical Practice Guidelines by the Endocrine Society do acknowledge body weight differentials and recommended obese subjects be given two to three times more vitamin D to satisfy their body's vitamin D requirement, however they acknowledge that there are no studies that clearly justify this [7], [20].

The objectives of the present study are to characterize the dose response relationship of oral vitamin D supplementation and serum 25(OH)D in a large sample of healthy volunteers, and to quantify the extent this dose response relationship is different for BMI and for absolute body weight. As the effect of vitamin D on serum calcium and the risk for hypercalcemia is the most common argument against high doses of vitamin D supplementation, we further studied the relationship between vitamin D supplementation and calcium homeostasis.

## Methods

This study is based on information from healthy volunteers participating in a preventive health program provided by the Pure North S'Energy Foundation (PN), a not-for-profit charitable organization providing free services since October 2007. The program and data collection protocol are described elsewhere [21], [22]. In brief, PN offers health promotion counseling with an emphasis on vitamin D supplementation as their volunteers mostly reside in the Canadian province of Alberta which is located between the 49^th^ and 60^th^ parallel north. PN asks participants to complete a lifestyle questionnaire, have their height and weight measured, have a medical history, and have blood drawn for the assessment of serum 25(OH)D. Since January 2009 the medical history recorded the question ‘how much vitamin D supplementation are you using?’ This includes vitamin D from vitamin D supplementation and from multivitamins. Also calcium supplementation was recorded in the medical history. All 22,214 per protocol study visits, that are typically scheduled once a year, prior to June 2013 were included in the present study. All 25(OH)D measurements were assessed with an automated chemiluminescent immunoassay from DiaSorin (LIAISON) which measures the combination of D2 and D3 and which has coefficients of variation ranging from 6.8% to 8.8%.

Various shapes of the relationship between vitamin D intake and 25(OH)D have been proposed [11], [23]–[26]. These include linear, polynominal, bi-phasic, exponential and ‘exponential plus linear’ relationships [11], [23]-[26]. We therefore sought to identify the regression model that best characterized the relationship by comparing linear, quadratic, cubic, linear-log, exponential and ‘exponential plus linear’ regression models on the basis of the Akaike Information Criteria (AIC) [27].

We examined the importance of both BMI and absolute body weight for the dose response relationship of oral vitamin D supplementation and serum 25(OH)D while adjusting for the confounding potential of age, sex and season using multivariable regression models. We compared BMI and absolute body weight both as continuous and categorical variables. When categorized, individuals with a BMI of less or equal than 18.5, more than 18.5 and less or equal than 25, more than 25 and less or equal than 30, and more than 30 were considered underweight, normal weight, overweight and obese, respectively [28]. Weight was categorized as less than 60 kg, 60 kg to 80 kg, more than 80 kg and less or equal to 100 kg, and more than 100 kg.

Assessments of height and weight were missing in 3% of the assessments. These records were excluded in analyses with BMI and absolute body weight as continuous covariates, but were included in analyses with BMI and absolute body weight as categorical covariates by considering missing values as a missing category. Differences in the dose response relationship of oral vitamin D supplementation and serum 25(OH)D by BMI were visualized through plots of model estimated 25(OH)D levels for any given supplementation levels.

As the available data included repeated observations for a subset of 3416 (19.4%) subjects, we included a random intercept in all the exponential regression models. The effects of vitamin D supplementation on calcium levels and probability of hypercalcemia were analyzed using linear regression and logistic regression, respectively.

All analyses were conducted using SAS 9.4 (SAS Institute, Cary NC) and the dose response curves were fitted using PROC NLMIXED, a SAS procedure for fitting nonlinear mixed effect models. Statistical significance was defined as p-values less than 0.05.

PN anonymized their data prior to forwarding it to the University of Alberta for analyses. The Human Research Ethics Board of the University of Alberta had approved access to and analysis of the PN data for the purpose of the present analyses.

## Results

Participants reported vitamin D supplementation ranging from 0 to 55,000 IU per day. Sixty-nine participants (0.3%) reported supplementation above 20,000 IU per day. The participants had serum 25(OH)D levels ranging from 10.1 to 394 nmol/L. Of all participants, 33.4% were normal weight, 1.3% underweight, 36.9% overweight and 28.4% obese (Table 1).

**Table 1:** Summary of 22,214 simultaneous assessments of oral vitamin D supplementation and serum 25(OH)D level.

	*N*	*%*	*Mean*	*Std*
Vitamin D supplementation (IU per day)	22214		2841.6	4022.5
Serum 25(OH)D level nmol/L	22214		90.5	46.5
Albumin corrected calcium (mmol/L)	10940		2.4	0.1
**Age (Years)**				
<40	7800	35.1		
40 to 49	4766	21.5		
50 to 59	5291	23.8		
60+	4357	19.6		
**Gender**				
Female	10944	49.3		
Male	11270	50.7		
**Weight Status**				
Underweight	279	1.3		
Normal weight	7197	33.4		
Overweight	7962	36.9		
Obesity	6131	28.4		
**Absolute weight**				
<60 kg	2270	10.6		
60 kg to 80 kg	8734	40.8		
80.1 to 100 kg	7232	33.8		
>100 kg	3158	14.8		
**season**				
Winter	7320	33.0		
Spring	7039	31.7		
Summer	4294	19.3		
Fall	3561	16.0		

Table 2 depicts characteristics of the linear, quadratic, cubic, linear-log, exponential and ‘exponential plus linear’ regression models that describe the dose response relationship between oral vitamin D supplementation and serum 25(OH)D. The exponential regression model appeared to describe the dose response relationship best. This conclusion is based on the observation that the AIC for the exponential regression model was lower than for of the other regression models (Table 2).

**Table 2:** The relationship between oral vitamin D supplementation and serum 25(OH)D levels estimated with six different parametric regression models.

	*Linear*		*Quadratic*		*Cubic*		*Linear-log*		*Exponential*		*Exponential plus Linear*	
*Parameter*	*β (95%CI)*	*p-value*	*β (95%CI)*	*p-value*	*β (95%CI)*	*p-value*	*β (95%CI)*	*p-value*	*β (95%CI)*	*p-value*	*β (95%CI)*	*p-value*
Intercept (Y0)	104.1(100.9,107.2)	<.001	101.8(98.7,105.0)	<.001	101.1(97.9,104.2)	<.001	18.3(14.5,22.1)	<.001	100.7(97.5,103.8)	<.001	100.8(97.6,103.9)	<.001
Age	0.3(0.3,0.3)	<.001	0.3(0.2,0.3)	<.001	0.3(0.2,0.3)	<.001	0.2(0.1,0.2)	<.001	0.2(0.2,0.3)	<.001	0.2(0.2,0.3)	<.001
BMI	−1.6(−1.7,−1.5)	<.001	−1.6(−1.7,−1.5)	<.001	−1.6(−1.7,−1.5)	<.001	−1.5(−1.6,−1.4)	<.001	−1.5(−1.6,−1.4)	<.001	−1.5(−1.6,−1.4)	<.001
Sex (male vs female)	−4.6(−5.7,−3.6)	<.001	−4.4(−5.4, −3.3)	<.001	−4.2(−5.2, −3.1)	<.001	−2.7(−3.8, −1.7)	<.001	−4.0(−5.1, −3.0)	<.001	−4.1(−5.1, −3.0)	<.001
**Season**												
Spring	1.4(0.2,2.7)	0.023	0.9(−0.3,2.1)	0.142	0.9(−0.3,2.1)	0.132	2.1(0.9,3.3)	<.001	1.0(−0.2,2.2)	0.105	1.0(−0.2,2.2)	0.116
Summer	4.1(2.7,5.5)	<.001	4.3(2.9,5.7)	<.001	4.5(3.1,5.9)	<.001	5.3(3.9,6.8)	<.001	4.6(3.2,6.0)	<.001	4.6(3.2,6.0)	<.001
Fall	2.5(1.0,4.1)	<.001	3.0(1.5,4.5)	<.001	3.1(1.6,4.6)	<.001	3.3(1.8,4.8)	<.001	3.1(1.6,4.6)	<.001	3.1(1.6,4.6)	<.001
Winter	ref.		ref.		ref.		ref.		ref.		ref.	
Vitamin D daily dose (per 1000 IU)	5.9(5.7,6.0)	<.001	8.5(8.2,8.7)	<.001	9.6(9.2,10.0)	<.001					−1.1(−2.9,0.8)	0.256
Vitamin D daily dose (per 1000 IU)^2^			−0.2(−0.2, −0.2)	<.001	−0.3(−0.4, −0.3)	<.001						
Vitamin D daily dose (per 1000 IU)^3^					0.0(0.0,0.0)	<.001						
log10 Vitamin D (per 1000 IU)							34.2(33.4,34.9)	<.001				
A									100.0(94.0,105.9)	<.001	132.1(71.1,193.2)	<.001
B									0.1(0.1,0.1)	<.001	0.1(0.1,0.1)	<.001
**AIC**	**217424.1**		**216795.0**		**216736.3**		**217725.2**		**216721.8**		**216722.1**	

β: β-coefficient; 95% CI: 95% confidence Interval; ref: reference category; AIC: Akaike Information Criteria; A and B are parameters in the exponential and ‘exponential plus linear’ regression models. In the exponential model, Y = Y0+A*(1-e^-BX^), Y denotes serum 25(OH)D, Y0 (intercept) denotes serum 25(OH)D in the absence of vitamin D supplementation, and X denotes vitamin D supplementation. The six parametric regression models are compared on the basis of the AIC. The parametric regression model with the lowest AIC value (the exponential model) is the model that best describes the observations.

**Figure 1:**
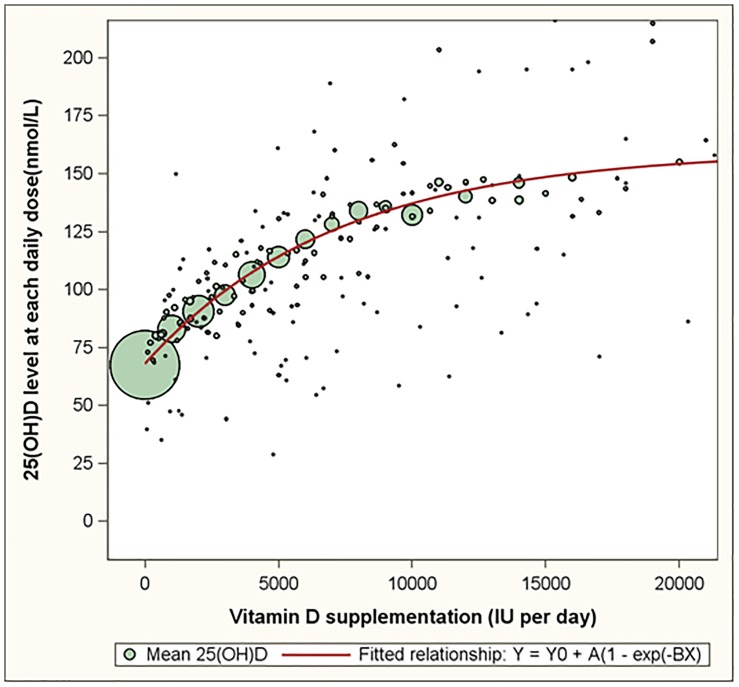
The dose response relationship between oral vitamin D supplementation and serum 25(OH)D levels based on 22,214 observations of healthy volunteers. Footnote: Bubbles represent the mean plasma 25(OH)D level for all reported daily doses. The size of the bubbles is proportional to the number of assessments for each of the reported daily doses. The red line represents the fitted dose response curve.

The dose response relationship between vitamin D supplementation and serum 25(OH)D for supplementation levels of 20,000 IU per day or less is depicted in Figure 1. Bubbles represent the mean serum 25(OH)D level for all reported doses of vitamin D supplementation. The size of the bubbles is proportional to the number of assessments for each of the doses. Though the entire range of supplementation values was included in analyses, the graphs are plotted up to 20,000 IU. The red line represents the fitted exponential dose response curve and confirms the clear impression from the bubbles that the dose response relationship is non-linear and levels off at increasingly higher supplementation levels. The parameter Y0, that represents the average serum 25(OH)D level reached without vitamin D supplementation, was estimated to be 68.0 nmol/L (95% CI: 67.3, 68.7).

**Figure 2:**
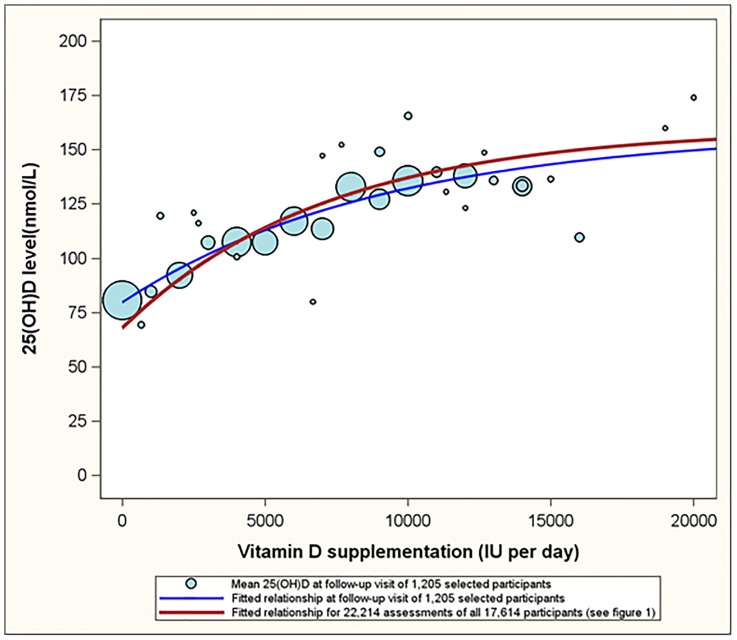
The relationship between oral vitamin D supplementation dose and serum 25(OH)D level at the follow up visits of a subgroup of 1205 healthy volunteers who reportedly did not supplement at their baseline visit. Footnote: Bubbles represent the mean plasma 25(OH)D level for all reported daily doses. The size of the bubbles is proportional to the number of assessments for each of the reported daily doses. The blue line represents the fitted relationship for the subgroup of participants who reportedly did not supplement at their baseline visit. The red line represents the fitted relationship of the entire sample (22,214 observations). Both analytic approaches revealed similar dose response relationships as the red and blue lines are similar.

For Figure 2 we restricted our analyses to those subjects that had both their baseline visit and a follow up visit between January 2009 and June 2013 and had reported not to supplement with vitamin D at their baseline visit (1205 subjects, 2410 assessments). This analysis mimics a pre-post comparison of an intervention: a comparison of observations prior to introduction to vitamin D supplementation with observations, on average, 0.98 years after the baseline visit. As such, the blue bubbles in figure 2 represent the expected 25(OH)D level of participants who have been taken oral doses of vitamin D for an average of 0.98 year since baseline. The blue line represents the fitted dose response curve for this subset of 1205 subjects. The red line is identical to the red line in Figure 1 representing the fitted dose response curve for the complete sample (22,214 assessments from 17,614 subjects). The red line in Figure 2 (and the observations presented in Figure 1) could be described as a ‘snapshot of an ongoing intervention program’. The fact that the red and blue lines in Figure 2 are similar illustrates that a ‘pre-post comparison’ and a ‘snapshot of an ongoing intervention program’ reveal similar results.

Both Figure 1 and 2 show that the increase in serum 25(OH)D is leveling off at higher doses of vitamin D supplementation. Serum 25(OH)D levels are estimated to increase on average by 11.98 nmol/L per 1,000 IU in the supplementation interval of 0 to 1,000 IU per day and by 1.13 nmol/L per 1,000 IU in the supplementation of 15,000 to 20,000 IU per day. In addition to supplementation, also age, BMI, absolute body weight, gender and season are associated with serum 25(OH)D levels in a statistically significant manner (Table 3). The differences across BMI categories (Table 3, column 1) are pronounced: obese subjects and overweight subjects had serum 25(OH)D levels that were on average 19.8 nmol/L lower and 8.0 nmol/L lower than those of normal weight subjects, respectively. The differences in serum 25(OH)D levels between underweight and normal weight subjects were not statistically significant. Differences across absolute weight categories were also substantial and statistically significant (Table 3, column 2). In table 3, the AIC values were smaller in models that included BMI relative to models that included absolute body weight regardless of whether they were considered as categorical (213710.6 versus 213990.2; Table 3: columns 1 and 2) or continuous (213602.0 versus 213852.0; Table 3, columns 1 and 2) covariates, suggesting BMI to be a better predictor of 25(OH)D relative to absolute weight. When considering BMI and absolute weight simultaneously (Table 3: columns 3 and 6), BMI appeared to be the better proximate determinant of 25(OH)D. This conclusion is based on the observation that the estimated coefficients for BMI changed only slightly when absolute weight is included in the model (column 3 versus column 1 and column 6 versus column 4), while the coefficients for absolute weight changed substantially when BMI is included (Table 3, column 3 versus 2 and column 6 versus 5).

**Table 3:** Importance of body mass index and absolute body weight for the relationship between oral vitamin D supplementation and serum 25(OH)D.

	*Categorical BMI and absolute weight*						*Continuous BMI and absolute weight*					
	*Column 1*		*Column 2*		*Column 3*		*Column 4*		*Column 5*		*Column 6*	
	*BMI*		*Weight*		*combined*		*BMI*		*Weight*		*Combined*	
	*β (95%CI)*	*p-value*	*β (95%CI)*	*p-value*	*β (95%CI)*	*p-value*	*β (95%CI)*	*p-value*	*β (95%CI)*	*p-value*	*β (95%CI)*	*p-value*
BMI (continuous)							−1.5(−1.6, −1.4)	<.001			−1.9(−2.2, −1.7)	<.001
Absolute weight (continuous)									−0.4(−0.5, −0.4)	<.001	0.1(0.1,0.2)	<.001
												
**BMI category**												
Underweight	1.1(−3.4,5.7)	0.623			0.7(−3.9,5.2)	0.774						
Normal weight	Ref.				Ref.							
Overweight	−8.0(−9.3, −6.7)	<.001			−7.4(−8.9, −6.0)	<.001						
Obesity	−19.8(−21.1, −18.4)	<.001			−18.8(−20.9, −16.8)	<.001						
**Absolute weight category**												
<60 kg			8.9(7.1,10.7)	<.001	3.8(1.9,5.7)	<.001						
60 kg to 80 kg			Ref.		Ref.							
80.1 to 100 kg			−6.7(−7.9, −5.4)	<.001	1.0(−0.6,2.5)	0.223						
>100 kg			−14.8(−16.4, −3.1)	<.001	−0.7(−3.0,1.6)	0.561						
												
**AIC**	**213710.6**		**213990.2**				**213602.0**		**213852.0**			

Footnote: β: β-coefficient; 95% CI: 95% confidence Interval; ref: reference category; AIC: Akaike Information Criteria; All the estimates are adjusted for age, gender, season and vitamin D supplementation; AIC values are based on models fitted with observations for which both BMI and absolute body weight were not missing.

**Figure 3:**
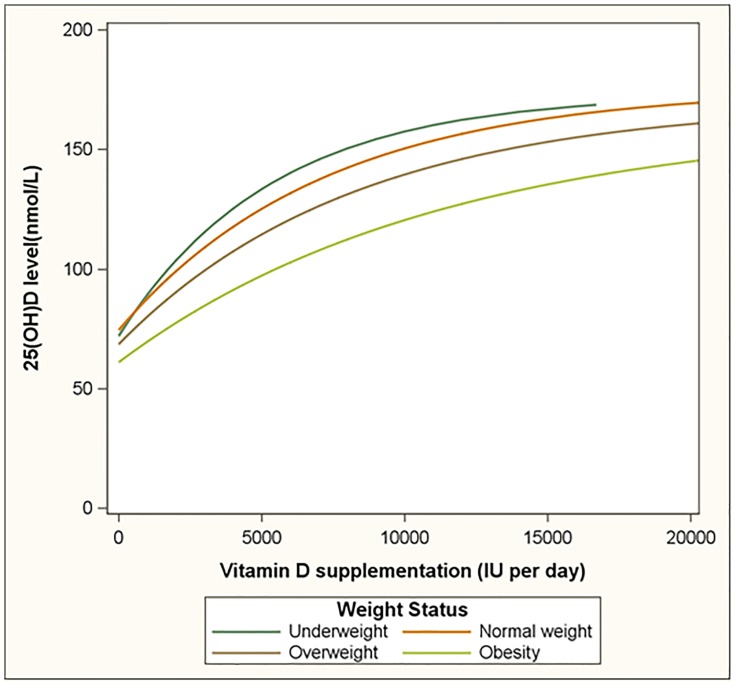
The dose response relationship between oral vitamin D supplementation and plasma 25(OH)D levels by body mass index category. Footnote: the lines are estimated using an exponential plus linear regression model that adjusted for age, gender, and season.

The BMI differences in the dose response relationship are further visualized in Figure 3. Relative to normal weight subjects, obese subjects had lower 25(OH)D values and curved differently. Serum 25(OH)D was estimated to increase at an average rate of 13.1 nmol/L per 1000 IU, 11.5 nmol/L per 1000 IU and 8.6 nmol/L per 1000 IU among normal weight, overweight and obese participant, respectively in the supplementation interval of 0 to 1000 IU per day. The average rates of increase then reduce to 1.3 nmol/L per 1000 IU, 1.5 nmol/L per 1000 IU and 1.9 nmol/L per 1000 IU, respectively in the supplementation interval of 15,000 IU to 20,000 IU per day.

Table 4 provides estimates for the relationship of supplementation and serum 25(OH)D by BMI category. Supplementation with 600 IU per day would achieve average serum 25(OH)D levels of 83, 76 and 66 nmol/L for normal weight, overweight and obese participants, respectively (Table 4). Average serum 25(OH)D levels of 100 nmol/L in normal weight, overweight and obese subgroups, are estimated to require supplementation with 2,080 IU, 3,065 IU and 5,473 IU per day, respectively (Table 4). Relative to normal weight participants, this represents a 1.47 and 2.6 times higher dose for overweight and obese subjects, respectively.

**Table 4:** Estimated average serum 25(OH)D levels for various vitamin D supplementation doses and the estimated average vitamin D supplementation doses for various serum 25(OH)D levels for normal weight, overweight and obese individuals.

	*Underweight*		*Normal weight*		*Overweight*		*Obesity*	
	*Est.*	*95% CI*	*Est.*	*95% CI*	*Est.*	*95% CI*	*Est.*	*95% CI*
***Vitamin D supplementation dose in IU per day***	***Serum 25(OH)D level in nmol/L***							
600	83	(78,88)	83	(82,84)	76	(75,77)	66	(65,67)
1000	89	(85,94)	88	(87,89)	80	(79,81)	70	(69,71)
2000	104	(98,109)	99	(98,100)	90	(89,91)	78	(77,79)
4000	125	(117,133)	118	(116,119)	107	(106,109)	91	(90,93)
10000	158	(151,164)	151	(149,152)	140	(138,141)	121	(119,122)
15000	167	(161,173)	163	(160,166)	153	(151,156)	135	(133,138)
***Serum 25(OH)D level in nmol/L***	***Vitamin D supplementation dose in IU per day***							
75	151	(-,507)	28	(-,115)	534	(450,619)	1663	(1538,1790)
100	1723	(1360,2113)	2080	(1978,2183)	3065	(2928,3204)	5473	(5190,5763)
125	3959	(3142,4923)	4964	(4763,5172)	6733	(6470,7005)	11272	(10701,11874)
150	7871	(6380,9945)	9858	(9389,10360)	13501	(12700,14389)	—	
								

Footnote: Est: Estimated average; 95% CI: 95% Confidence interval; —: estimate for vitamin D supplementation dose is above 20,000 IU a day.

**Figure 4:**
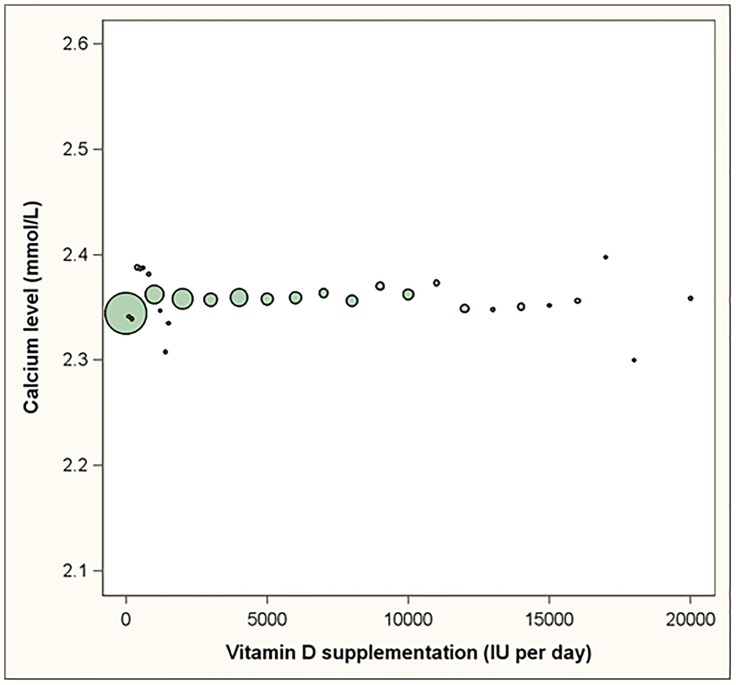
The dose response relationship between vitamin D supplementation and calcium levels. Footnote: Bubbles represent the mean serum calcium level for all reported daily doses. The size of the bubbles is proportional to the number of assessments for each of the reported daily doses. The linear regression line is adjusted for age, gender, BMI, season and calcium supplementation.

For the 10,940 visits that included assessments for serum calcium, the mean albumin corrected calcium level was 2.35 mmol/L (standard deviation = 0.11) and ranged from 1.79 to 3.23. Figure 4 shows the dose response relationship between vitamin D supplementation and serum calcium levels. In a linear regression model that adjust for age, BMI, gender, season, and calcium supplementation, serum calcium levels did not increase significantly by increasing daily vitamin D supplementation: 0.001 mmol/L per 1000 IU increase in daily vitamin D supplementation, p-value = 0.165 (Table 5).

**Table 5:** Determinants of serum calcium and hypercalcemia based on 10,940 assessments from healthy volunteers.

	*Serum calcium level*		*Hypercalcemia*	
	*β (95%CI)*	*p-value*	*OR (95%CI)*	*p-value*
Vitamin D daily dose (per 1000 IU)	0.001(-0.000,0.001)	0.165	0.97(0.91,1.03)	0.286
Calcium supplementation (per 100 mg)	0.003(0.001,0.006)	0.015	1.20(1.03,1.39)	0.023
**Age (Years)**				
<40	ref.		ref.	
40 to 49	0.003(−0.003,0.009)	0.302	2.09(1.09,4.00)	0.026
50 to 59	0.029(0.023,0.035)	<.001	3.20(1.80,5.67)	<.001
60+	0.044(0.038,0.050)	<.001	5.73(3.32,9.87)	<.001
**Gender**				
Male	−0.019(−0.024, −0.015)	<.001	0.56(0.39,0.80)	0.001
Female	ref.		ref.	
**Weight Status**				
Underweight	−0.005(−0.026,0.015)	0.609	1.16(0.28,4.82)	0.837
Normal weight	ref.		ref.	
Overweight	−0.003(−0.009,0.002)	0.239	1.05(0.69,1.59)	0.834
Obesity	0.008(0.002,0.013)	0.009	1.27(0.83,1.93)	0.267
**Season**				
Spring	−0.024(−0.031, −0.016)	<.001	0.92(0.58,1.47)	0.728
Summer	−0.017(−0.022, −0.011)	<.001	0.57(0.34,0.96)	0.034
Fall	−0.021(−0.027, −0.015)	<.001	0.91(0.59,1.42)	0.682
Winter	ref.		ref.	

Footnote: β: β-coefficient; OR: odds ratio; 95% CI: 95% confidence Interval; ref: reference category; all estimates are adjusted for age, gender, BMI category, season, and supplementation with vitamin D and calcium.

Of the 10,940 visits that included assessments of serum calcium, 189 (1.7%) had albumin corrected calcium levels exceeding 2.6 mmol/L (hypercalcemia). In a logistic regression model that adjusted for age, BMI, gender, season, and calcium supplementation, there was no statistically significant effect of vitamin D supplementation on the probability of having hypercalcemia (Table 5: Odds ratio = 0.97 per 1000 IU increase in daily vitamin D supplementation, p-value = 0.286). Also, the probability of having hypercalcemia was not statistical significantly different for overweight and obese subjects relative to normal weight subjects. In contrast, female gender and older age appeared important risk factors for hypercalcemia (Table 5).

## Discussion

We observed substantial differences in serum 25(OH)D across categories of BMI and absolute body weight, which concurs with observations by others [11]–[19] and deviates from reports that concluded an absence of body weight differentials [29], [30]). The present study suggests that, on statistical grounds, BMI is the better measure relative to absolute body weight to determine which vitamin D doses are needed for which body weight groups to achieve specific serum 25(OH)D targets. The present study also adds to the existing knowledge by revealing that the magnitude of the differences in serum 25(OH)D between normal weight and obese subjects varies by supplementation dose. Furthermore, this study provides detailed recommendations for supplementation to achieve 25(OH)D targets specific for normal weight, overweight and obese individuals (provided in Table 4). These recommendations appeared 2 to 3 times higher for obese participants relative to normal weight subjects, depending on the 25(OH)D target level. This is consistent with the Endocrine Society's recommendation that obese subjects be given two to three times more vitamin D [7], [20]. Estimates for overweight individuals appeared approximately 1.5 times higher relative to normal weight subjects. The number of underweight participants was relatively small though do suggest underweight subjects need less vitamin D supplementation relative to normal weight subjects. Others had reported an absence of differences between underweight and normal weight subjects [31]. This study recommends guidelines for vitamin D supplementation be specific for normal weight, overweight and obese individuals, but this study does not recommend specific supplementation levels or specific 25(OH)D target levels.

We observed an exponential dose response relationship whereby serum 25(OH)D levels off with increasing levels of oral vitamin D supplementation. On average, serum 25(OH)D was estimated to increase by approximately 12.0 nmol/L per 1,000 IU in the supplementation interval 0 to 1,000 IU per day and by 1.1 nmol/L per 1,000 IU in the supplementation interval of 15,000 to 20,000 IU per day. Other studies reported that an additional 1,000 IU of vitamin D could increase serum 25(OH)D by approximately 20 to 25 nmol/L [24], [32]. The substantial differences may arise from their focus on subjects with low baseline serum 25(OH)D levels, whereas our study had enrolled healthy volunteers. Also Garland et al. [23] and Aloia et al. [11] had reported dose response relationships that leveled off. Garland et al. [23] modeled cross sectional observations of 3,667 US based community volunteers and Aloia et al. [11] plotted aggregated outcomes of 62 controlled trials. The increase in serum levels per unit increase in supplementation varied across the three studies as a result of, at least in part, differences in study population characteristics. Participants of the present study resided at, on average, a latitude of 53 degrees [21] and had presumably less subcutaneous production of vitamin D by sun exposure. Participants of the present study reportedly without supplemental vitamin D had an average serum level of 68 nmol/L, which approximates the Canadian average of 67.7 nmol/L [33]. Luxwolda et al [34] reported serum 25(OH)D levels ranging from 58 to 171 nmol/L (average 115 nmol/L with 90% having serum 25(OH)D of less than 150 nmol/L) for traditional living populations in East Africa and suggested that this may represent ‘natural levels’. The present study shows that on average the upper limit of 171 nmol/L was not reached with oral supplementation of 20,000 IU per day.

The IOM report states that vitamin D toxicity is rare at 10,000 IU per day but more common with regular doses of 50,000 IU per day, suggesting the toxicity range likely starts at 500 nmol/L [8]. In the present study where substantial numbers of participants reported up to 20,000 IU of vitamin D per day, and some even more, the highest serum 25(OH)D value observed was 394 nmol/L. This seems consistent with safety studies that reported an absence of adverse effects from vitamin D doses of up to 50,000 IU per day [35], [36]. Our observation that supplementation dose was not associated with hypercalcemia in a statistically significant manner is consistent with an earlier report that daily doses of up to 40,000 IU per day are not associated with hypercalcemia [37].

This study represents the first body weight specific characterization of the dose response relationship of a wide range of vitamin D supplementation and serum 25(OH)D levels. The strengths of the study include the large population, the relatively high supplementation dose, and the fact that all serum samples were subjected to the same 25(OH)D assessment methods, with heights and weights measured rather that self-reported. The questions regarding vitamin D supplementation by health professionals may have introduced recall bias and social desirability bias. Information on the duration of using vitamin D supplementation had not been collected. Where participants changed their doses in the months prior to the 25(OH)D assessment, this also may have introduced error. However, we expect the latter error to be small as all participants are aware that the objective of the 25(OH)D assessment is to receive advice on vitamin D supplementation dose, and therefore not likely to changing their supplementation dose in the months prior to the assessment. Although this study included residents of Northern latitude where sun exposure and subcutaneous synthesis of vitamin D are considered limited, and despite our adjustment for season as a proxy of sun exposure, we acknowledge that a precise measure of daily hours of sun exposure may have yielded better estimates. Likewise, where we did adjust for the confounding potential of age, gender, and season, we acknowledge that further adjustment for skin color, physical activity, outdoor activities and dietary intake may have yielded better estimates. Unlike in blinded trials, confounding by indication, whereby participants whose 25(OH)D levels respond well to vitamin D may lower their dose and participants whose 25(OH)D levels do not respond well may increase their dose, may have biased the estimates of the present study. Lastly, we recommend large randomized controlled trails among healthy subjects be analyzed on BMI differentials in the dose response relationship between vitamin D intake and serum 25(OH)D to confirm the present findings.

In summary, we recommend clinical guidelines for vitamin D supplementation be specific for normal weight, overweight and obese individuals. In this study we provide body weight specific recommendations to reach certain serum 25(OH)D target levels.

## References

[1] Cranney A, Horsley T, O'Donnell S, Weiler H, Puil L, et al.. (2007) Effectiveness and safety of vitamin D in relation to bone health. Evid Rep Technol Assess (Full Rep) 1–235. PMC4781354 18088161

[2] Ebeling PR (2014) Vitamin D and bone health: Epidemiologic studies. Bonekey Rep 3: 511. 24818003 10.1038/bonekey.2014.6 PMC4015454

[3] Holick MF (2007) Vitamin D deficiency. N Engl J Med 357: 266–281. 17634462 10.1056/NEJMra070553

[4] Sanabria A, Dominguez LC, Vega V, Osorio C, Duarte D (2011) Cost-effectiveness analysis regarding postoperative administration of vitamin-D and calcium after thyroidectomy to prevent hypocalcaemia. Rev Salud Publica (Bogota) 13: 804–813. 22634947 10.1590/s0124-00642011000500009

[5] Winzenberg T, Jones G (2013) Vitamin D and bone health in childhood and adolescence. Calcif Tissue Int 92: 140–150. 22710658 10.1007/s00223-012-9615-4

[6] Health Canada. Vitamin D and Calcium: Updated Dietary Reference Intakes. Available: http://www.hc-sc.gc.ca/fn-an/nutrition/vitamin/vita-d-eng.php. Accessed 2014 Jan 25.

[7] Holick MF, Binkley NC, Bischoff-Ferrari HA, Gordon CM, Hanley DA, et al (2011) Evaluation, treatment, and prevention of vitamin D deficiency: an Endocrine Society clinical practice guideline. J Clin Endocrinol Metab 96: 1911–1930. 21646368 10.1210/jc.2011-0385

[8] Institute of Medicine (2011) Dietary Reference Intakes for Calcium and Vitamin D. The National Academies Press. 21796828

[9] Heaney RP (2011) Serum 25-hydroxyvitamin D is a reliable indicator of vitamin D status. Am J Clin Nutr 94: 619-20; author reply 620. 10.3945/ajcn.111.019539 21775574

[10] Holick MF (2010) Vitamin D: Physiology, Molecular Biology, and Clinical Applications. Humana Press.

[11] Aloia JF, Patel M, Dimaano R, Li-Ng M, Talwar SA, et al (2008) Vitamin D intake to attain a desired serum 25-hydroxyvitamin D concentration. Am J Clin Nutr 87: 1952–1958. 18541590 10.1093/ajcn/87.6.1952

[12] Blum M, Dallal GE, Dawson-Hughes B (2008) Body size and serum 25 hydroxy vitamin D response to oral supplements in healthy older adults. J Am Coll Nutr 27: 274–279. 18689559 10.1080/07315724.2008.10719700 PMC2729752

[13] Chao YS, Brunel L, Faris P, Veugelers PJ (2013) The importance of dose, frequency and duration of vitamin D supplementation for plasma 25-hydroxyvitamin D. Nutrients. 5: 4067–4078. 10.3390/nu5104067 PMC3820059 24152747

[14] Drincic A, Fuller E, Heaney RP, Armas LA (2013) 25-hydroxyvitamin d response to graded vitamin d3 supplementation among obese adults. J Clin Endocrinol Metab 98: 4845–4851. 24037880 10.1210/jc.2012-4103

[15] Gallagher JC, Sai A, Templin T, Smith L (2012) Dose response to vitamin D supplementation in postmenopausal women: a randomized trial. Ann Intern Med 156: 425–437. 22431675 10.7326/0003-4819-156-6-201203200-00005

[16] Gallagher JC, Jindal P, Lynette MS (2013) Vitamin D does not Increase Calcium Absorption in Young Women: A Randomized Clinical Trial. J Bone Miner Res. 10.1002/jbmr.2121 24166866

[17] Lee P, Greenfield JR, Seibel MJ, Eisman JA (2009) Center JR (2009) Adequacy of vitamin D replacement in severe deficiency is dependent on body mass index. Am J Med 122: 1056–1060. 19854337 10.1016/j.amjmed.2009.06.008

[18] Tepper S, Shahar DR, Geva D, Ish-Shalom S (2013) Predictors of serum 25(Oh)D increase following bimonthly supplementation with 100,000IU vitamin D in healthy, men aged 25–65 years. J Steroid Biochem Mol Biol. 10.1016/j.jsbmb.2013.12.005 24333798

[19] Zittermann A, Ernst JB, Gummert JF, Borgermann J (2013) Vitamin D supplementation, body weight and human serum 25-hydroxyvitamin D response: a systematic review. Eur J Nutr. 10.1007/s00394-013-0634-3 24292820

[20] Holick MF, Binkley NC, Bischoff-Ferrari HA, Gordon CM, Hanley DA, et al (2012) Guidelines for preventing and treating vitamin D deficiency and insufficiency revisited. J Clin Endocrinol Metab 97: 1153–1158. 22442274 10.1210/jc.2011-2601

[21] Chao YS, Brunel L, Faris P, Veugelers PJ (2013) Vitamin D status of Canadians employed in northern latitudes. Occup Med (Lond) 63: 485–493. 24027218 10.1093/occmed/kqt106

[22] Heaney RP, French CB, Nguyen S, Ferreira M, Baggerly LL, et al (2013) A novel approach localizes the association of vitamin D status with insulin resistance to one region of the 25-hydroxyvitamin D continuum. Adv Nutr 4: 303–310. 23674796 10.3945/an.113.003731 PMC3650499

[23] Garland CF, French CB, Baggerly LL, Heaney RP (2011) Vitamin D supplement doses and serum 25-hydroxyvitamin D in the range associated with cancer prevention. Anticancer Res 31: 607–611. 21378345

[24] Heaney RP, Davies KM, Chen TC, Holick MF, Barger-Lux MJ (2003) Human serum 25-hydroxycholecalciferol response to extended oral dosing with cholecalciferol. Am J Clin Nutr 77: 204–210. 12499343 10.1093/ajcn/77.1.204

[25] Vieth R (2005). The pharmacology of vitamin D, including fortification strategies. In FD, GF, & PJ (Eds.), *Vitamin D* (pp. 995–1015).

[26] Vieth R (1999) Vitamin D supplementation, 25-hydroxyvitamin D concentrations, and safety. Am J Clin Nutr 69: 842–856. 10232622 10.1093/ajcn/69.5.842

[27] Akaike H (1973) Information theory as an extension of the maximum likelihood principle. In: Petrov BN, Csaki F. editors. Second International Symposium on Information Theory. Budapest. pp. 267–281.

[28] World Health Organization (2006) BMI Classification: The International Classification of adult underweight, overweight and obesity according to BMI.

[29] Nelson ML, Blum JM, Hollis BW, Rosen C, Sullivan SS (2009) Supplements of 20 microg/d cholecalciferol optimized serum 25-hydroxyvitamin D concentrations in 80% of premenopausal women in winter. J Nutr 139: 540–546. 19158226 10.3945/jn.108.096180

[30] Shab-Bidar S, Bours SP, Geusens PP, van der Velde RY, Janssen MJ, et al (2013) Suboptimal effect of different vitamin D3 supplementations and doses adapted to baseline serum 25(OH)D on achieved 25(OH)D levels in patients with a recent fracture: a prospective observational study. Eur J Endocrinol 169: 597–604. 23959785 10.1530/EJE-13-0068

[31] Divasta AD, Feldman HA, Brown JN, Giancaterino C, Holick MF, et al (2011) Bioavailability of vitamin D in malnourished adolescents with anorexia nervosa. J Clin Endocrinol Metab 96: 2575–2580. 21632810 10.1210/jc.2011-0243 PMC3146790

[32] Vieth R (2006) Critique of the considerations for establishing the tolerable upper intake level for vitamin D: critical need for revision upwards. J Nutr 136: 1117–1122. 16549491 10.1093/jn/136.4.1117

[33] Langlois K, Greene-Finestone L, Little J, Hidiroglou N, Whiting S (2010) Vitamin D status of Canadians as measured in the 2007 to 2009 Canadian Health Measures Survey. Health Rep 21: 47–55. 20426226

[34] Luxwolda MF, Kuipers RS, Kema IP, Dijck-Brouwer DA, Muskiet FA (2012) Traditionally living populations in East Africa have a mean serum 25-hydroxyvitamin D concentration of 115 nmol/l. Br J Nutr 108: 1557–1561. 22264449 10.1017/S0007114511007161

[35] Bischoff-Ferrari HA, Shao A, Dawson-Hughes B, Hathcock J, Giovannucci E, et al (2010) Benefit-risk assessment of vitamin D supplementation. Osteoporos Int 21: 1121–1132. 19957164 10.1007/s00198-009-1119-3 PMC3062161

[36] Hathcock JN, Shao A, Vieth R, Heaney R (2007) Risk assessment for vitamin D. Am J Clin Nutr. 85: 6–18. 10.1093/ajcn/85.1.6 17209171

[37] Cianferotti L, Marcocci C (2012) Subclinical vitamin D deficiency. Best Pract Res Clin Endocrinol Metab 26: 523–537. 22863394 10.1016/j.beem.2011.12.007

